# Squid male alternative reproductive tactics are determined by birth date

**DOI:** 10.1098/rspb.2024.0156

**Published:** 2024-04-24

**Authors:** Shota Hosono, Yoshio Masuda, Shun Tokioka, Tomohiko Kawamura, Yoko Iwata

**Affiliations:** ^1^ Atmosphere and Ocean Research Institute, The University of Tokyo, Kashiwa, Chiba 277-0882, Japan; ^2^ Miyagi Prefecture Fisheries Technology Institute, Ishinomaki, Miyagi, Japan; ^3^ Shiogama Field Station, Fisheries Resources Institute, Japan Fisheries Research and Education Agency, Shiogama, Miyagi, Japan

**Keywords:** alternative reproductive tactics, birth date hypothesis, spear squid, statolith, *Heterololigo bleekeri*

## Abstract

Alternative reproductive tactics (ARTs) are discontinuous phenotypes associated with reproduction, observed in males of many species. Typically, large males adopt a tactic of competing with rivals for mating, while small males adopt a tactic of stealing fertilization opportunities from the large males. The ‘birth date hypothesis', proposing that the date of birth influences the determination of each male's reproductive tactic, has been tested only in teleost fish to date. Here, the birth date hypothesis was tested in ARTs of Japanese spear squid *Heterololigo bleekeri* (consort/sneaker) by analysing statolith growth increments. The birth date significantly differed between consorts (early-hatched) and sneakers (late-hatched). However, no differences were detected in growth history up to 100 days from hatching. Most immature males caught during the reproductive season were larger than sneakers, and their hatch date was similar to that of consorts, suggesting that these immature males had already been following a life-history pathway as a consort. These results indicate that ARTs of *H. bleekeri* are determined based on their hatch date in early life. This study firstly suggests that the birth date hypothesis applies to aquatic invertebrates, suggesting that the mechanism by which birth date determines the individual phenotype is a phenomenon more common than previously believed.

## 1. Background

Intrasexual polymorphism within a single population is commonly observed in various taxa. In particular, males of many species exhibit alternative reproductive tactics (ARTs), observable as discontinuous phenotypic variation associated with reproduction, to maximize their reproductive success [1,2]. ARTs generally arise in the context of male–male competition for access to a mate and/or fertilization, with large males tending to adopt bourgeois tactics to guard and monopolize females and/or reproductive resources, while small males adopt parasitic tactics by stealing some of the fertilization success from large males [3–5]. Individuals of each reproductive tactic exhibit, accordingly, specific behaviour, morphology, physiology and timing of reproduction.

Mechanisms by which the reproductive tactic of each male is determined vary among species. Genetic polymorphisms resulting in ARTs have been demonstrated in species of fish [6], birds [7], damselflies [8] and isopods [9] but are considered rare, while ARTs determined by environmental factors are considered to be more common. When individuals can switch their reproductive tactics during their life, the tactic is determined based on their circumstances or their status at that time (e.g. sequential ARTs from a parasitic tactic to a bourgeois tactic [10]). However, if switching is not possible once initially determined (i.e. fixed ARTs), the tactic for each individual must be determined before the reproductive season. In some species, it is known that the allotted tactic is determined by the environment experienced in early life [11]. Clarifying the particular mechanisms underlying the determination of ARTs is crucial to understanding how the alternative phenotypes have evolved and are maintained within a population.

The ‘birth date hypothesis' has been proposed as one mechanism of ART determination [12]. In a species that has a relatively long breeding season during which it experiences seasonal environmental changes, early- and late-born individuals experience markedly different growth conditions, such as duration of the growth period and the environment experienced in early life. Therefore, the difference in birth date can result in different body sizes at maturity with consequences affecting ARTs. In the grass goby *Zosterisessor ophiocephalus* [13] and peacock blenny *Salaria pavo* [14], early- and late-born individuals differ in the duration of their growth period prior to the following breeding season, and therefore have a difference in body size at the beginning of the breeding season which governs their respective adoption of either the bourgeois or parasitic tactic. Welsh *et al*. [15] also demonstrated a birth date effect on the choice of reproductive tactics in smallmouth bass *Micropterus dolomieu*. However, so far the birth date hypothesis has been tested only in fish; no other taxonomic group.

Loliginid squids exhibit body size-associated male ARTs in which large bourgeois males, called ‘consort’ males, physically compete with other males and copulate with a female in a preferred mating position; and small parasitic males, called ‘sneaker’ males, avoid fights with other males but steal opportunities to perform a furtive style of copulation with females guarded by a consort [16–18]. Loliginid species are terminal spawners with a 1-year life span and their growth rate is strongly dependent on the environment experienced, including food availability and temperature [19,20]. Given these features, a few months difference in birth date is expected to make a considerable difference in subsequent life history in this group. In some loliginid species, such as *Doryteuthis plei* and *Uroteuthis edulis*, age analysis using statolith growth increments has revealed a difference in hatch date between individuals using the two ARTs [21,22]. However, it has been proposed that *D. plei* individuals change their reproductive tactics ontogenetically from sneaker to consort [21,23]. Even if such a species does indeed have only a single life-history pathway (i.e. all individuals become a sneaker at a younger age and a consort later), the difference in hatch date between tactics can be observed between consorts and sneakers (i.e. old consorts and young sneakers at the timing of capture have early and late birth dates, respectively). Information on ARTs of *U. edulis* is sparse and the possibility of ontogenetic change of tactics as in *D. plei* cannot be discounted. Therefore, although differences in hatch date were observed between ARTs, it is difficult to be certain whether or not there is a birth date effect on the determination of ARTs in these two species.

The Japanese spear squid *Heterololigo bleekeri* (Keferstein, 1866) is an ideal species to evaluate the birth date effect on ART determination in loliginid squid, since it is one of the most frequently studied cephalopods for research into ARTs [17,24–26]. In this species, ARTs are considered to be fixed, unlike those of *D. plei* [27]. Furthermore, since *H. bleekeri* is widely distributed, extending to the highest latitude of any loliginid squid [28], it has a well-defined but long reproductive season (from January to May) and experiences seasonal changes in the environment [29], resulting in marked differences in growth conditions in its early life history between early- and late-hatched individuals. These environment-related characteristics suggest the possibility that birth date determines ARTs in this species.

In the present study, to test the birth date hypothesis in *H. bleekeri*, the hatch date of consort and sneaker males was investigated by age analysis based on statolith growth increments in individuals caught at various times during the reproductive season. The growth rates between ARTs were compared by reconstructing the growth history of each individual based on its statolith growth increment widths, to investigate when the body size dimorphism begins and whether growth affects ART determination. In addition to consorts and sneakers, immature males were examined to determine whether or not male ARTs are fixed at an early stage of the life cycle dependent on the hatch date. In addition, the age of mature females was also analysed, which could be helpful to discuss a general relationship between the age and life-history traits in *H. bleekeri*.

## 2. Methods

For age analysis, *H. bleekeri* individuals were collected from commercially fished catches taken by inshore setnet or bottom trawl off Miyagi Prefecture, northeastern Honshu, Japan, during the reproductive season (January–May) in 2021 (on 18 and 26 January, 24 February, 8 March, 22 April and 10 May). A wet weight of 20 kg of squid was randomly collected on each sampling date. For back-calculation of the past mantle length (ML) at a certain age, we constructed a function between ML and statolith radius (SR; see below). To broaden the size range of individuals, squid collected by inshore setnet were used in addition to the above samples: in 2020 (on 21 August and 17 November), 2021 (on 23 December) and 2022 (on 18 January, 8 February, 14 March, 25 April and 16 May) and juvenile squid caught by inshore setnet on 28 July 2023 and by bottom trawl conducted by RV Wakataka Maru off Miyagi on 18 and 19 June, 2023.

In the laboratory, ML was measured to the nearest 1 mm and maturity stages were categorized based on the three stages reported for *D. plei* [30]. Squid males transfer sperm to females by spermatophores (capsules containing sperm). During mating, on exposure to seawater the spermatophore undergoes an everting reaction (the ‘spermatophoric reaction’) to form a spermatangium, which bears small spines that attach the spermatangium to the female [25]. Males were classified for their reproductive tactic based on spermatophore dimorphism: in consorts, the spermatophores are longer and produce rope-like spermatangia; sneakers have shorter spermatophores with drop-shaped spermatangia [25]. All males without developed spermatophores were pooled as ‘immature males’ for further analysis (as it is impossible to identify the subsequent reproductive tactic adopted until males are fully mature with spermatophores present in Needham's sac). Only mature females were analysed in the age analysis; immature females were not used. The sex and reproductive tactics of juvenile squid could not be determined.

Both statoliths (sense organs of equilibrium, analogous to fish otoliths [31]) were removed from each specimen, washed in distilled water, dried, and stored in a microtube until processing. In the loliginid squid species investigated to date, the statolith bears a thick deposit (called a ‘hatching check’) marking the day of hatching [32] and subsequently there is one growth increment per day [33], enabling the age of the individual to be estimated by counting the number of layers laid exterior to the hatching check.

**Figure 1.**
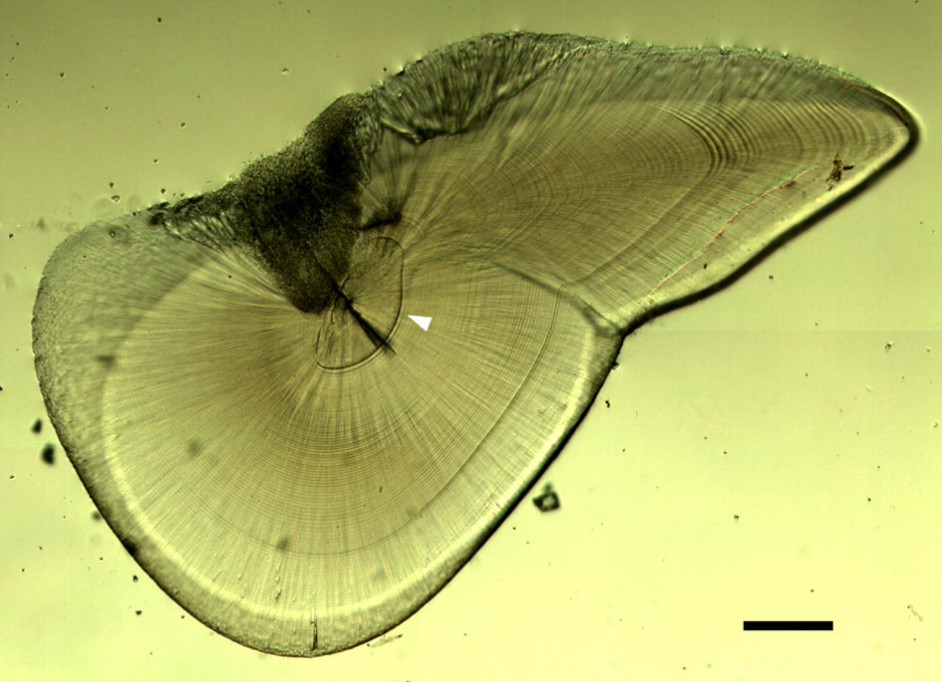
Light micrograph of a statolith section of *Heterololigo bleekeri* (scale bar: 100 µm). Arrowhead indicates the dark ‘hatching check’ increment.

A total of 201 males (97 consorts, 38 sneakers and 66 immature males) and 68 mature females were subjected to age analysis. Statoliths were ground from both convex and concave sides with wet waterproof sandpapers (grit nos. 1000, 2000, 4000; 3M). The core was exposed from the concave side and the statolith surface was polished with an active oxide polishing suspension (OP-S suspension, 0.25 µm; Struers) to improve the visibility of statolith microstructure (figure 1). The growth increments along the rostrum axis within the statolith were blind counted three times by a single reader at least 1 day apart under a light microscope at ×200 and ×500 magnification using an otolith increment analysis system (Ratoc System Engineering Co. Ltd, Tokyo, Japan). To test the accuracy of the three blind counts, the average per cent error (APE; [34]) and the coefficient of variation (CV; [35]) were calculated for each sample. If both APE and CV were lower than 10%, the counts were regarded as satisfactorily precise and the mean of the three counts (rounded down to the nearest integer) was used as the age of the individual [21]. The hatch date of each individual was calculated by subtracting the statolith-determined age from the date on which the individual was caught.

In total, 92 consorts and 35 sneakers (individuals analysed for their age) were used for back-calculation of past ML at a certain age (some individuals were excluded due to insufficient clarity of growth increments for the growth analysis). A relationship was obtained between ML and the distance from the core of the statolith to the edge, along the maximum growth axis in the rostrum (referred to as statolith radius; SR). An additional 23 consorts, 20 sneakers, 16 immature males, and 38 juveniles were added to this analysis to increase the sample size and to include the entire size range of the life history. This ML–SR relationship allows for back-calculation of the past ML of each individual at a given age by measuring accumulated growth increment width at that age, which corresponds to the SR at that age. The ML–SR relationship was significantly affected by reproductive tactics (regression analysis, *p* < 0.05). Since the sex and reproductive tactic of juveniles could not be identified, subsequent analyses were conducted separately for the group of consorts with immatures and for sneakers with immatures. Segmented linear regression was applied to the logarithmic ML–SR relationship for each tactic to obtain two regression lines separated at the switch point (consort, ln(SR) = 6.683; sneaker, ln(SR) = 6.845), respectively (consort first regression line, ln(ML) = 1.9877ln(SR) – 9.0182; consort second regression line, ln(ML) = 3.0440ln(SR) – 16.0780; sneaker first regression line, ln(ML) = 1.9948ln(SR) – 9.0608; sneaker second regression line, ln(ML) = 2.7567ln(SR) – 14.2760; electronic supplementary material, figure S1).

Increment widths were measured along the growth axis from the core to edge for each statolith to estimate the SR at each age (i.e. accumulated increment width at age). It was difficult to measure the increment widths along the growth axis for the second half of the life history (indistinct), making it impossible to estimate the past SR throughout the entire life history, but measurements included at least 100 days of age for all individuals. For back-calculation of ML at the *i*-th day, we used a combination of the body proportional (BP) method [36] and a modified Fry method [37]. When *SR_i_* was larger than the switch point, *ML_i_* was calculated using the log transformed BP calculation based on the second regression line of each tactic. When *SR_i_* was smaller than the switch point, firstly the ML at the switch point $\left(ML_{sp}\right)$ was calculated by the BP method and the value obtained was inserted into the modified Fry model, which was applied to the power function derived from the first regression line of each tactic. That is, when *SR_i_* ≥ switch point,

$$ML_{i}=\exp\left\{\left(\frac{\ln(ML_{c})}{v\ln(SR_{c})+u}\right)(v\ln(SR_{i})+u)\right\},$$

and when *SR_i_* < switch point,

$$ML_{i}=a+\exp\left\{\ln(ML_{0}-a)+\frac{\left(\ln(ML_{sp}-a)-\ln(ML_{0}-a))(\ln(SR_{i})-\ln(SR_{0})\right)}{\ln(SR_{sp})-\ln(SR_{0})}\right\},$$

where *ML* is mantle length, *SR* is statolith radius, and *v* and *u* are, respectively, the slope and intercept of the second regression line of each tactic. The subscripts *i*, *c*, *sp*, and 0 represent the age at the *i*-th day, the time of capture, the time of the switch point, and the time of hatching (biological intercept), respectively. *ML*_0_ was set as the mean size (3.13 mm; unpublished data) of the 93 hatchlings of *H. bleekeri*, and *SR*_0_ was set as the mean size of the hatching check of individuals for which SR was measured, which was 114.01 µm. The constant ‘*a*’ was calculated for each tactic according to [37] (consort, 2.0949; sneaker, 2.1195). Reconstructed ML at each age and the ML growth increment every 20 days were compared between the reproductive tactics.

For comparison of age among consorts, sneakers and mature females, a non-parametric Mann–Whitney *U*-test was used because the sample number was small on some catch dates. Statistical analysis for sneakers on 8 March, 2021, could not be conducted because no sneaker males were obtained on that day. The hatch dates of consorts, sneakers and immature males were compared with Student's *t*-test after testing for normality with the Shapiro–Wilk test and variance homogeneity with Bartlett's test. Differences in reconstructed ML and the growth amount between tactics were also examined using Student's *t*-test, but the Mann–Whitney *U*-test was used for growth during 80–100 days of age because these data lacked normality. We conducted a logistic regression in mature males to estimate the probability of adopting either tactic associated with the hatch date. We also performed multivariable logistic regression analysis in individuals subjected to growth analysis to assess the effect of hatch dates and growth in early life on the determination of reproductive tactics. For this analysis, hatch date and growth of each individual, including growth during 0–20, 20–40, 40–60, 60–80, 80–100 days of age, were used as independent variables, which were standardized by subtracting the mean and dividing by the standard deviation to facilitate comparison of the variable at different scales. We calculated the variance inflation factor (VIF) to address multicollinearity and found that all VIF values were at an acceptable level (maximum 5.8). All statistical analyses were conducted using R 4.3.2 [38].

## 3. Results

The values of APE and CV calculated from the three blind counts were lower than 10% in all analysed individuals, and the means were 2.45% (standard deviation 1.10%, maximum 4.61%) and 3.34% (standard deviation 1.49%, maximum 6.14%), respectively. These values indicated that the counts of growth increments were sufficiently accurate.

**Figure 2.**
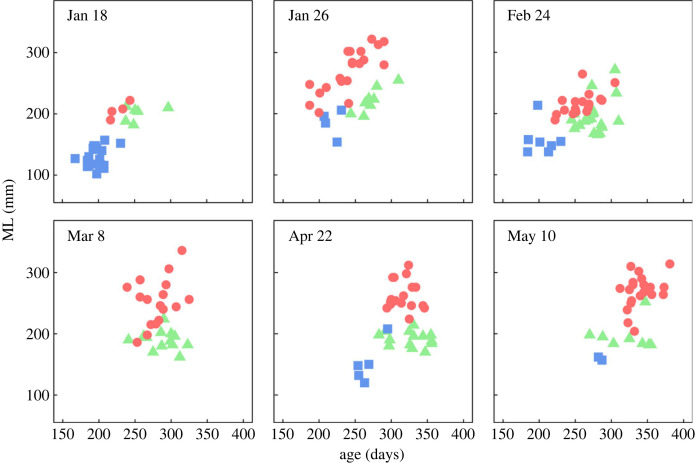
Relationship between mantle length (ML) and age in days on each catch date for consorts (red circles, *n* = 4, 20, 17, 17, 19, and 20 in chronological order), sneakers (blue squares, *n* = 20, 4, 7, 0, 5 and 2), and mature females (green triangles, *n* = 6, 8, 19, 13, 14 and 8).

Estimated ages were 187–381 days for consorts, 167–295 days for sneakers and 237–356 days for mature females, which is in agreement with *H. bleekeri* as a terminal spawner with a 1-year life span (figure 2). Sampled individuals tended to be older as the reproductive season (i.e. catch dates) progressed in all groups, but there was no trend of increase in ML. For most catch dates, sneakers were significantly younger than consorts and mature females (electronic supplementary material, table S1). The age of mature females was higher than that of consorts until 24 February, but no difference was observed after 8 March (electronic supplementary material, table S1).

**Figure 3.**
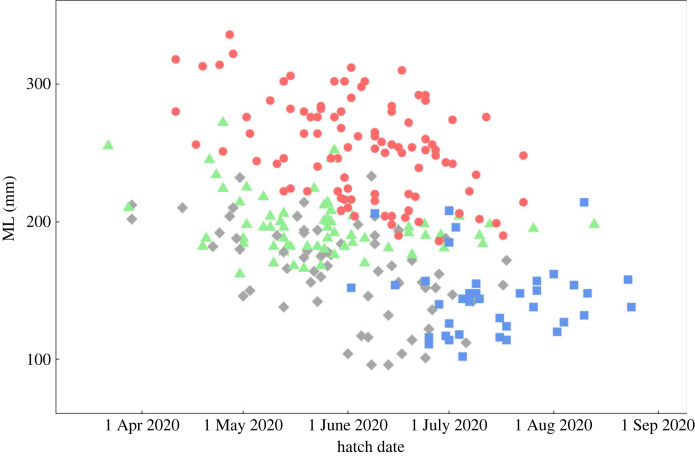
Relationship between mantle length (ML) and hatch date for consorts (red circles), sneakers (blue squares), mature females (green triangles) and immature males (grey diamonds).

**Figure 4.**
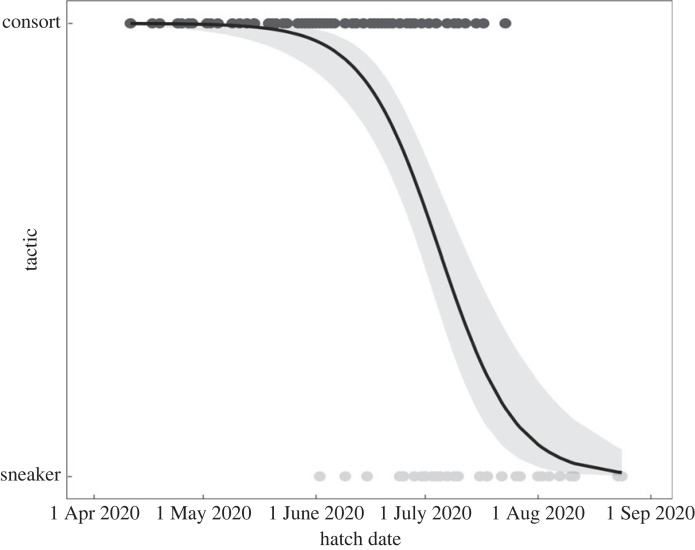
Logistic regression between hatch date and probability to be either tactic. Grey band indicates 95% confidence interval.

**Figure 5.**
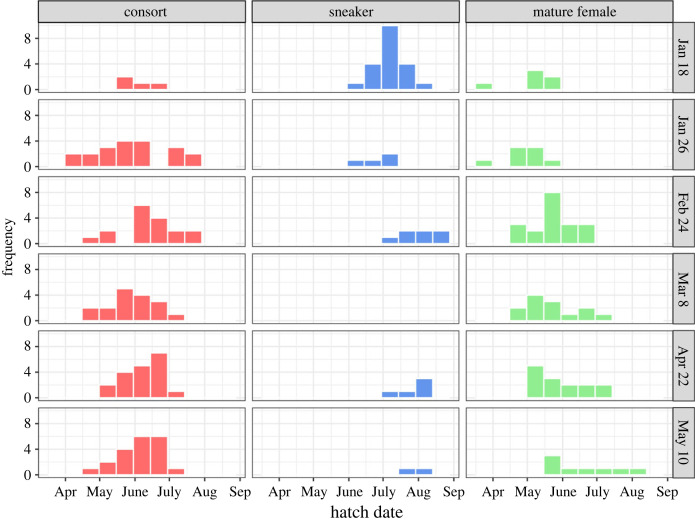
Hatch date distribution for consorts, sneakers, and mature females on each catch date.

The hatch date of consorts, back-calculated from the catch date and age at capture, was significantly earlier than that of sneakers (consorts had hatched from 11 April to 23 July, mean date 4 June; sneakers had hatched from 2 June to 24 August, mean date 14 July; *t*_133_ = −9.32, *p* < 0.01; figure 3). The range of hatch dates for each reproductive tactic overlapped between 2 June and 23 July. A logistic regression analysis demonstrated that the hatch date had a significant effect on male tactic (*z* = 5.596, *p* < 0.01; figure 4). The date distinguishing the tactics with a probability of 50% was 4 July. The range of hatch dates of mature females overlapped with those of both consort and sneaker males (figure 3). The hatch date of consorts did not differ among the catch dates. However, the sneakers and mature females who reproduced late in the reproductive season tended to have hatched later (figure 5).

In this study, immature males were present in the samples collected until the middle of the reproductive season (8 March). Their hatch date, ranging from 29 March to 18 July, did not differ significantly from that of consorts (*t*_161_ = 1.46, *p* = 0.15) but it was significantly earlier than that of sneakers (*t*_102_ = 9.53, *p* < 0.01; figure 3). Furthermore, 74% of immature males had a larger ML than the mean ML of sneakers (ML 145.1 mm) and also hatched earlier than 4 July, which is estimated by logistic regression as the date determining the male tactic with a 50% probability. However, these immature males had not reached the mean body size of consorts (which was 253.4 mm).

**Figure 6.**
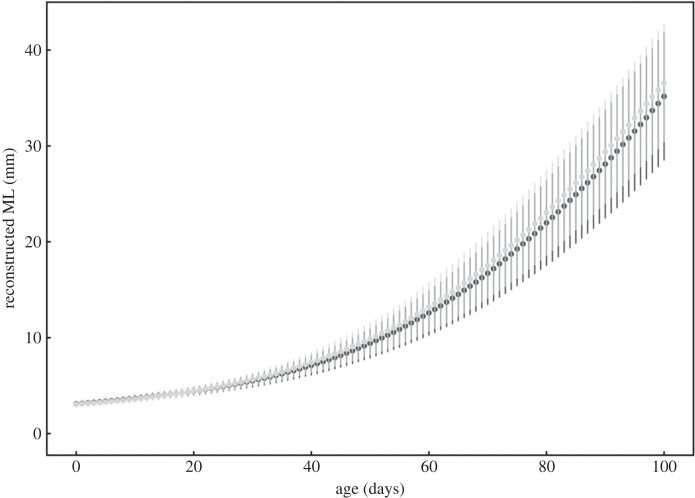
Reconstructed mantle length (ML) of each age up to 100 days. Each point represents the mean reconstructed ML of each reproductive tactic at each day, with vertical lines indicating the standard deviation. Dark grey represents consort males; light grey, sneaker males.

**Figure 7.**
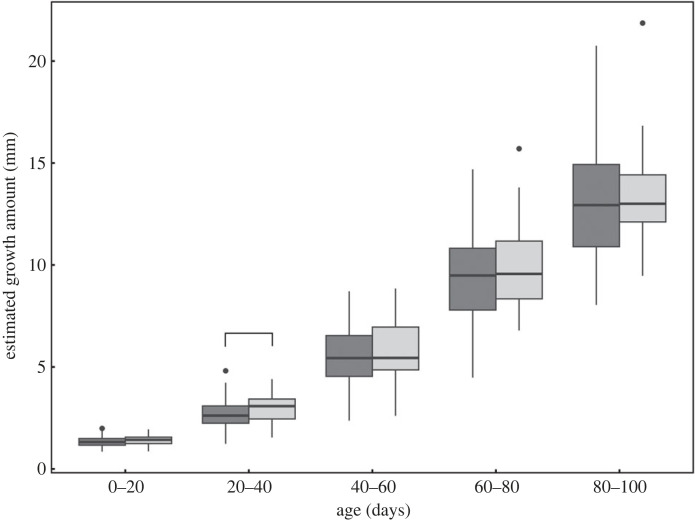
Estimated growth amount every 20 days of age. Growth amount at 20–40 days of age was significantly different between consorts (dark grey) and sneakers (light grey).

Figure 6 shows the trend of mean back-calculated ML at each age up to 100 days for consorts and sneakers. The back-calculation of ML in this study was not markedly different from the growth pattern observed in experimental culture of *H. bleekeri* hatchlings in captivity [39], indicating that the reconstruction of ML was reliable. In this age range, there were no differences in the estimated ML between reproductive tactics at all ages (*t*-test, *p* > 0.05). The growth increment during days 20–40 was significantly larger for sneakers than consorts (*t*_125_ = −2.09, *p* < 0.05), but not different during 0–20 (*t*_125_ = −1.52), 40–60 (*t*_125_ = −1.02), 60–80 (*t*_125_ = −0.99), and 80–100 days (*U* = 1496, *p* > 0.05 for all age periods, figure 7). The multivariable logistic regression, incorporating hatch date and growth increment every 20 days as the variable, showed a significant effect of hatch date on the determination of reproductive tactics (table 1). Although growth at 20–40 days differed between tactics, the model suggested that growth during early life history did not significantly affect the determination of reproductive tactics.

**Table 1.** The results of multivariable logistic regression analysis.

variable	coefficient	standard error	odds ratio (95% CI)	*p*-value
hatch date	−0.10803	0.02159	0.90 (0.85–0.93)	>0.01
growth 0–20 days	0.43994	0.46881	1.55 (0.63–4.01)	0.35
growth 20–40 days	−1.12645	0.65407	0.32 (0.084–1.13)	0.09
growth 40–60 days	1.11331	0.6805	3.04 (0.84–12.51)	0.10
growth 60–80 days	0.20206	0.59752	1.22 (0.38–4.045)	0.74
growth 80–100 days	0.03095	0.37615	1.03 (0.49–2.17)	0.93

## 4. Discussion

In the present study, birth date was found to have a significant influence on determining the ARTs of *H. bleekeri*: early-hatched males tended to become consorts and late-hatched males tended to become sneakers. This result suggests that these tactics are not simply inherited from paternal tactics, but rather are likely to be determined by a combination of physical, biological and social environmental factors related to hatch date. Because female squid store sperm from both consort and sneaker males throughout the breeding season [40] and use a mixture of sperm from both sources to fertilize their eggs during a spawning event [17], it is expected that offspring descended from both types of sire hatch throughout the hatching season (from March to August in the present study). If ARTs were based on polymorphic genotypes, the hatch date would not be different between tactics (i.e. early-hatched sneakers should be observed as well as consorts). Therefore, the ARTs of *H. bleekeri* are determined by differing environmental cues associated with the different hatch dates.

The results for immature males suggest that the ARTs of *H. bleekeri* are determined early in the life history and do not change once determined; that is, *H. bleekeri* ARTs are fixed. Although sampling for the age analysis was conducted throughout the reproductive season of *H. bleekeri*, immature males appeared until the middle of the reproductive season (8 March 2021). Most of these immature males were of sufficient body size to mature as a sneaker male (i.e. their ML was larger than the mean ML of sneaker males), and their hatch dates were not different from those of consort males but earlier than those of sneakers. These results suggested that these immature males had already followed the life-history pathway as a consort male but had yet to mature. If the ARTs had been determined based on their condition or circumstances at the time of the reproductive season, these immature males should have expressed the sneaker tactic due to insufficient body size to be a consort male. Furthermore, if they initially mature as a sneaker and switch to a consort tactic as they grow up, immature males larger than sneakers would not be observed during the reproductive season. Hirohashi *et al.* [27] presumed the ARTs of *H. bleekeri* to be fixed because their reproductive traits are clearly dimorphic and no intermediate males were identified, unlike in *D. plei* [21,23]. Our result, indicating that most immature males had probably already determined their life-history pathway as a result of their hatch date, provides more direct support for this hypothesis. The immature males had probably postponed maturation in favour of growth to achieve sufficient body size to become a consort and compete with rival consort males. Similarly, non-reproductive males have been observed during the breeding season in the Mediterranean wrasse *Symphodus ocellatus*, which are known to then adopt the bourgeois tactic later [10,41]. The trade-off between maturation and somatic growth has been confirmed in an octopus, *Octopus hummelincki* [42], so it is presumed that remaining in a non-reproductive mode is a strategic advantage to maintain a high growth rate and become larger (and, for squid, become a consort). However, it remains unclear whether such a trade-off exists in *H. bleekeri*.

The difference in hatch date can influence the choice of reproductive tactic by exposing each individual to different growth conditions, such as growth period and environment experienced in early life [12]. This birth date hypothesis has been tested for several species of teleost fish [13–15], where it was shown that early-born individuals have a longer time to grow until the beginning of the breeding season than late-born individuals, resulting in differences in body size and therefore reproductive tactics. The same mechanism possibly explains ART determination in *H. bleekeri*. If body size at the beginning of the reproductive season depends on the growth period from the hatch date, early-hatched individuals will have a higher likelihood of exceeding the size threshold and becoming a consort male (even if they exceed the threshold, maturation may be delayed, when their size would be insufficient as a consort and they would remain as a non-reproductive male until they had grown even larger). However, late-hatched individuals cannot exceed the size threshold to become a consort because the duration of growth is too short, so instead they become a sneaker male. This is the most common mechanism supporting the birth date hypothesis, and would occur in various animal taxa not only in fish.

The difference in hatch date also results in differences in the environmental conditions which each individual experiences in early life and may influence the growth trajectory and subsequent mating tactic. However, back-calculated ML up to 100 days (approximately ML 35 mm) did not show any significant differences between reproductive tactics. This result suggested that the difference in body size between tactics increased after 100 days. Stomach content analysis has revealed that the feeding habits of *H. bleekeri* depend on body size [43]. Up to 90 mm ML, they feed mainly on crustaceans, the proportion of fish content increases up to 200 mm ML, and beyond that size they prey mainly on fish. This indicates that expected growth increases with a larger body size in the later stages of the life history, particularly beyond 90 mm ML. Therefore, it is possible that consorts become larger by accelerating their growth after reaching approximately 200 mm ML in association with a change in feeding habit, while sneakers maintain a small body size due to a more modest growth rate. Another possibility is that if growth increases exponentially towards the later stages of the life history, body size dimorphism between tactics arises due to differences in age. However, since there was an overlap in ages between consorts and sneakers, which have a large difference in body size (figure 2), the body size difference may have increased purely because of changes in growth rate beyond 100 days of age.

The growth increments in the early life stage also did not affect ART determination (although growth during 20–40 days of age was larger for sneakers than consorts). This is surprising because the growth rate of squid is sensitive to environmental factors, such as water temperature: many previous studies have shown that the water temperatures experienced during early life are important for growth in cephalopods [19,20]. In a mathematical simulation, Forsythe [44] found that, in *Loligo forbesi*, the body weight at 90 days after hatching approximately doubled with each 1°C increase in water temperature experienced, and this difference would affect even size at maturity. Sea surface temperature (SST) around the sampling site in 2020 showed broad seasonal changes (electronic supplementary material, figure S2). Although the recorded temperatures may not reflect accurately the temperatures experienced by the individuals used in the present study during their early life, they are indicative of overall seasonal changes in water temperature in the sampling area. Therefore, it was expected that individuals hatching in different seasons would exhibit a different growth pattern, but no significant differences were observed to be associated with ART determination. A slight positive correlation between hatch date and growth rate was observed (electronic supplementary material, figure S3), but it was not strong enough to solely determine growth patterns. Thus, the absence of differences in growth pattern between reproductive tactics is probably due to the complex interaction of various environmental factors other than water temperature.

Although the growth history early in life did not differ between reproductive tactics, it is expected that environmental differences experienced by early- and late-hatched individuals may have directly determined the reproductive tactics. For example, epigenetic modifications induced by environmental cues during a specific period in early life history can regulate DNA expression and cause a divergence in the life-history pathway, as observed in temperature-dependent sex determination [45], and would be applicable to intrasexual polymorphism. During the periods when the squid hatchlings were destined to become either all consorts or all sneakers, SST was increasing rapidly, whereas during the overlap period (when the hatchlings were not destined to become either consort or sneaker), SST was around 20°C and increasing more gradually (electronic supplementary material, figure S2). Such seasonality may act as a stimulus and cause a divergence in the life-history pathway. Furthermore, as loliginid squid have a planktonic phase and can be dispersed by currents in the early stages [46], mature males caught on the same sampling site may be a mixture of individuals hatched and grown in different areas. Therefore, it is highly plausible that some environmental cues determine the ARTs of squid early in the life history. To test this hypothesis, it would be useful to compare the water temperature experienced between reproductive tactics by measuring statolith Sr/Ca, which has a negative correlation with water temperature [47].

The range of hatch dates of consorts and sneakers overlapped between 2 June and 23 July, indicating that even individuals that have hatched during the same period expressed different reproductive tactics, and that individuals that hatched during this period experienced different environmental conditions. As mentioned above, individuals hatching in different locations can be in a single spawning aggregation due to the broad distribution potential of the planktonic stage of early life [46]. If the ARTs of *H. bleekeri* are determined by environmental factors experienced in early life, it is possible that individuals hatching at the same time but in different sites are exposed to different conditions and adopt different tactics at maturity. Additionally, short-timescale changes in the environment might affect the determination of ARTs in *H. bleekeri*. During the period when both consorts and sneakers hatched, SST increased relatively slowly but with large fluctuations (electronic supplementary material, figure S2). If individuals respond differently to such short-timescale fluctuations in environmental conditions, the overlap of hatch dates between consorts and sneakers is plausible.

It is also conceivable that individuals have experienced similar environments in their early life history but exhibited different tactics due to individual variations in thresholds for the environmental stimuli which determine tactics [48]. An environmental threshold model has been proposed in which genetic variation in reaction norms (the pattern of phenotypic expression of a single genotype to some specific environmental variable) among individuals can lead to different phenotypes even when individuals are exposed to the same environmental conditions [49]. If so, it is possible that individuals hatching between 2 June and 23 July experienced similar environments but followed different life-history pathways due to individual differences in their threshold for environmental stimuli.

Differences in the distribution of hatch dates among consorts, sneakers and mature females are attributed to their life-history traits related to sex and reproductive tactics. The sampled squids identified as sneakers and mature females were from later and later hatch dates as the reproductive season proceeded (figure 5), suggesting that newly matured individuals were continuously recruited into the spawning population and individuals that had joined the population previously were no longer alive. In females, since their reproductive success is largely defined by fecundity proportional to body size, they are expected to start maturing when they reach a certain body size which is optimized as the balance of benefit from growth and survival cost. For sneakers, since their body size should not affect their reproductive success (they do not compete with other males for mating), they are expected to start reproduction as soon as they reach the minimum body size at which they can mate with females. Therefore, the optimal strategy of females and sneakers is to join the spawning group as soon as they reach a certain body size. By contrast, the hatch date of consorts did not differ among the individuals sampled on different dates throughout the reproductive season. Since, unlike sneakers, consorts physically compete with rival males, their body size is critical for reproductive success. Consequently, individuals hatching early and following the life history pathway as a consort would delay maturity until their body size is sufficient to compete with rival males. The presence of large immature males appears to support this (i.e. these immature males may have been delaying their maturation in a non-reproductive state as a tactic to become a successful consort later in the season). Thus, the distribution of hatch dates of consorts would differ from that of sneakers and females due to their life-history traits, even when new recruits are arriving constantly.

In sneakers and mature females, late-hatched individuals tended to mature when older despite having a ML similar to that of early-hatched individuals, indicating that late-hatched individuals had experienced poorer daily growth. However, growth analysis of sneakers suggests that the difference in hatch date resulted in only minor variations in ML at 100 days of age (electronic supplementary material, figure S4). This indicates that the growth after 100 days varied based on hatch date among individuals even within sneakers and females. One possible cause of the variation in growth after 100 days is cannibalism. In loliginid squid, it is known that larger individuals frequently prey on smaller ones [50], and Natsukari & Tashiro [43] observed cephalopods in the diet of *H. bleekeri* of ML ≥ 45 mm. Late-hatched individuals of sneakers and females are expected to be relatively small within a population at a certain time, making them more susceptible to predation by relatively large early-hatched individuals. Therefore, if cannibalism enhances growth, early-hatched individuals have the potential advantage of accelerated growth by preying on relatively small late-hatched individuals. In this way, differences in relative body size at a given point in time can have a large influence on growth and subsequent life history.

In this study, we examined the age, hatch date and past growth of individuals collected throughout the distinct reproductive season of *H. bleekeri* and found that the birth date effect plays an important role in ART determination. The birth date hypothesis has been validated only in teleost fish until now, but this study has demonstrated the hypothesis in aquatic invertebrates for the first time. Differences in birth date occur in many animal taxa except for species with synchronized spawning, suggesting that the mechanism by which birth date determines the phenotype of each individual is a more common phenomenon than currently believed. In the present study, we also observed the growth history of *H. bleekeri*, but the environmental factors involved still remain to be determined, as do the ways in which they exert their influence based on hatch date to affect ART determination in *H. bleekeri*. In this species, distinct dimorphisms in reproductive traits, such as the form of spermatangia and sperm morphology, have been observed [25–27,51], and differences in gene expression have also been identified between the spermatozoa of consorts and sneakers [52]. Further epigenetic and endocrine studies to understand how the difference in hatch date influences the regulatory cascades underlying the phenotypic variation will help to further elucidate the mechanism of ART determination.

## Data Availability

Data used in this study are archived in the Dryad Digital Repository: https://doi.org/10.5061/dryad.r4xgxd2jw [53]. Supplementary material is available online [54].
